# The influence of electronic health record use on collaboration among medical specialties

**DOI:** 10.1186/s12913-020-05542-6

**Published:** 2020-07-22

**Authors:** Janita F. J. Vos, Albert Boonstra, Arjen Kooistra, Marc Seelen, Marjolein van Offenbeek

**Affiliations:** 1 Faculty of Economics and Business, University of Groningen, Groningen, The Netherlands; 2 Certe, Groningen, The Netherlands; 3 University Medical Center Groningen, Groningen, The Netherlands

**Keywords:** Electronic health records, Collaboration, Collaborative affordances, Use

## Abstract

**Background:**

One of the main objectives of Electronic Health Records (EHRs) is to enhance collaboration among healthcare professionals. However, our knowledge of how EHRs actually affect collaborative practices is limited. This study examines how an EHR facilitates and constrains collaboration in five outpatient clinics.

**Methods:**

We conducted an embedded case study at five outpatient clinics of a Dutch hospital that had implemented an organization-wide EHR. Data were collected through interviews with representatives of medical specialties, administration, nursing, and management. Documents were analyzed to contextualize these data. We examined the following collaborative affordances of EHRs: (1) portability, (2) co-located access, (3) shared overviews, (4) mutual awareness, (5) messaging, and (6) orchestrating.

**Results:**

Our findings demonstrate how an EHR will both facilitate and constrain collaboration among specialties and disciplines. Affordances that were inscribed in the system for collaboration purposes were not fully actualized in the hospital because:

(a) The EHR helps health professionals coordinate patient care on an informed basis at any time and in any place but only allows asynchronous patient record use.

(b) The comprehensive patient file affords joint clinical decision-making based on shared data, but specialty- and discipline-specific user-interfaces constrain mutual understanding of that data. Moreover, not all relevant information can be easily shared across specialties and outside the hospital.

(c) The reduced necessity for face-to-face communication saves time but is experienced as hindering collective responsibility for a smooth workflow.

(d) The EHR affords registration at the source and registration of activities through orders, but the heightened administrative burden for physicians and the strict authorization rules on inputting data constrain the flexible, multidisciplinary collaboration.

(e) While the EHR affords a complete overview, information overload occurs due to the parallel generation of individually owned notes and the high frequency of asynchronous communication through messages of varying clinical priority.

**Conclusions:**

For the optimal actualization of EHRs’ collaborative affordances in hospitals, coordinated use of these affordances by health professionals is a prerequisite. Such coordinated use requires organizational, technical, and behavioral adaptations. Suggestions for hospital-wide policies to enhance trust in both the EHR and in its coordinated use for effective collaboration are offered.

## Background

Collaboration among health professionals of various disciplines is considered a key factor in achieving high quality patient care [1, 2]. The symptoms of many of today’s patients, especially those with chronic diseases, are complex, and often require health professionals from different medical specialties to collaborate [3]. To collaborate effectively, it is necessary to share knowledge and skills, integrate information, and work as a cohesive health care team often while being in different locations [3, 4].

Electronic Health Records (EHRs) are implemented for various reasons, including to support coordination, collaboration, and shared decision- making, and are considered as a major means to deliver high-value care [5–8]. Physicians who have access to a patient’s health data through EHRs are able to immediately review the patient’s medical history, lab results, and other relevant information. However, EHRs have also been identified as constraining medical work [9], including collaboration [10–13]. Studies have addressed unanticipated problems such as alert fatigue [14], paper persistence [15], workflow mismatches [16] and time consuming system demands [17–19] resulting in reduced face-to-face patient care [20] making EHR-enabled collaboration troublesome and highly context dependent [21]. Given this situation, we are interested in how healthcare professionals interact and communicate, and eventually collaborate or are constrained in their collaboration as a result of the affordances offered by an EHR [11].

Affordances are potentials for behaviors that arise from the relationship between an artefact (here, an EHR) and goal-oriented actors (here, medical specialists) to achieve specific outcomes such as multidisciplinary collaboration [7]. In this paper, we label the affordances that are specifically inscribed in an EHR to facilitate or constrain collaboration as collaborative affordances. Recently, Bardram and Houben [22] demonstrated how EHRs provide the following four affordances for collaboration: Portability, Co-located Access, Shared Overview and Mutual Awareness.

Drawing on data from five multidisciplinary outpatient clinics of a Dutch hospital that had implemented a comprehensive EHR, this study examines how the collaborative affordances of an EHR facilitate or constrain actual collaboration.

### Collaboration in healthcare

The relevance of collaboration in healthcare is growing, and visions for more collaborative care are evident in both academic literature [2, 23, 24] and current practice [25]. We adopt the following definition of collaboration: *“a complex phenomenon that brings together two or more individuals, often from different professional disciplines, who work to achieve shared aims and objectives”* ([26], p.41].

Distinctions can be made in the degree of collaboration. First, *multidisciplinary* collaboration refers to healthcare professionals using the *“skills and experience of individuals from different disciplines, with each discipline approaching the patient from their own perspective”* ([27], p.1). Here, the disciplines work independently on discipline-specific care plans that are implemented together, but are not yet integrated into a single approach. Second, *interdisciplinary* collaboration integrates distinctive disciplinary approaches into a single consultation [27]. Healthcare professionals who work in an interdisciplinary way build on each other’s expertise and skills to obtain mutually defined goals [23]. However, how and how effectively healthcare professionals collaborate is influenced by their work context. Clear goals and rules, respect and trust between actors, clear organizational structures, and organizational support may all help effective collaboration [28].

Evidence shows that healthcare professionals can have divergent goals [29, 30]. Divergent goals in using an EHR can lead to non-aligned use patterns between collaborating professionals. Further, the established communication patterns within clinical departments can also result in divergent use of a hospital-wide EHR [31]. Moreover, some care professionals and departments may use an EHR in unstructured ways, and this could decrease the documentation quality [11]. A possible consequence of a lowered documentation quality could be reduced trust in the system, and this could constrain EHR-enabled collaboration among healthcare professionals from different departments. While an EHR can facilitate multidisciplinary collaboration during ward rounds, this depends on certain wider design issues being addressed, such as the social ergonomics of the devices involved, inclusion of paper records, and support in improving the technical system [32]. Data quality and accessibility issues have been found to threaten the EHR’s usefulness for multidisciplinary relationship-building, communicating, coordinating, and collaborative decision-making [10]. These authors argued that a multiplicity of communication channels, including the EHR-induced ones, may actually inhibit collaboration. Indeed, in another study, the introduction of an EHR increased documentation variability and limited collaboration between clinicians [33]. The complexity of healthcare practice, and demands for flexibility, may actually require EHRs that go beyond passive information storage and offer stronger support for collaboration [33]. To summarize, greater insight is required into the linkages between inter-professional communication patterns and the use of specific EHR affordances if one is to improve EHR integration in healthcare practices [10]. In the current paper, affordance theory is applied in unravelling these linkages.

### Theory of affordances

In this paper, we adopt the theory of affordances as a theoretical lens through which to understand the relationship linking information systems, actors, and use outcomes [34]. According to Gibson’s landmark definition [35], an affordance is what is offered, provided, or furnished to someone or something by an object. Within the information systems field, the basic principle of affordance theory is that information systems provide affordances for action possibilities, such as collaboration.

The theory of affordances views an information system in terms of what it affords or constrains actors to do [34, 36, 37]. These potentials for behaviors arise from the ability of a relationship between an artefact (e.g. an EHR) and goal-oriented actors (e.g. medical specialists) to achieve immediate concrete outcomes (e.g. inter-professional collaboration) [7]. Significantly, users first have to perceive these affordances and only then can they actualize them in order to effectively use an information system. As such, the theory takes the system into consideration but also recognizes the influence of actors. Affordances can exist without being actualized or even recognized by the actors: they only offer potential for action [35, 38, 39]. In other words, EHRs provide certain affordances that are built into the system by the EHR provider with specific actors in mind. However, the benefits that accrue from using these affordances depend on how these actors perceive and actualize them [7].

Strong et al. [7] specifically show that EHRs provide affordances to coordinate, monitor, standardize, and integrate care; capture, access, and use data about patients; substitute healthcare professionals, shift work across roles; and increasingly use information in clinical decision-making. More recently, Bardram and Houben [22] show that EHRs contain so-called collaborative affordances that enable collaborative action and workflow among different actors. In their study, they define collaborative affordances as *“a relation between an artifact and a set of human actors, that affords the opportunity for these actors to perform a collaborative action within a specific social context*” ([22], p. 8). The authors identify four collaborative affordances: portability (to navigate health records between locations), co-located access (to support simultaneous access), shared overview (to collectively build a shared information overview), and mutual awareness (to maintain mutual awareness of the work’s progress), but acknowledge that future research may identify further collaborative affordances.

EHRs’ affordances are not actualized in a vacuum: contextual factors influence how actors perceive and/or actualize them [39]. A hospital could, for example, organize improvement meetings to share ideas and stimulate affordance actualization across its departments [7]. In a remote telemedicine project in Nepal, the actualization of crucial affordances was found to depend on the accommodation of changes in personal, social, and cultural arrangements [40]. The latter study also points to the interdependency between different collaborative affordances: if actors actualize some affordances, this may lead to an outcome that may trigger the recognition of other affordances [40]. By drawing on data from five diverse multidisciplinary outpatient clinics in a hospital, we examined how an EHR’s inscribed collaborative affordances facilitated and constrained actual collaboration within and between disciplines and medical specialties.

## Methods

### Research site and department selection

To examine how an EHR’s collaborative affordances support or impede multidisciplinary collaboration between medical specialties within five outpatient clinics, we conducted an embedded case study in a hospital in the Netherlands. Adopting an embedded case-study approach allowed us to study EHR-facilitated collaboration in its natural setting and to recognize the complexities involved.

In the hospital investigated, a commercial cloud-based standard EHR system from a well-known global vendor had been implemented a year before the start of this study. The EHR implementation was also a response to the nationwide legislation prescribing to record patient data in organization wide Electronic Health Records. Before its implementation, departments used their own departmental IT applications and paper based systems to support their healthcare processes. After the EHR implementation, hospital-wide policies were developed to promote EHR use and the entering of patient data during the medical examination and visits. However, there were no formal sanctions for not using the EHR in line with the way intended by the implementers. Further, each clinic had its own management board, which had some discretion regarding the adoption and use of the EHR routines. The clinics researched were selected to give variation in the number of specialties involved, type of care, and the workflow dependencies between the specialties. Table 1 provides an overview of the breadth of the outpatient clinics and the functions of those we interviewed.

**Table 1:** Description of the selected outpatient clinics

Outpatient clinic	Number of specialties	Interviewed professionals (*n* = 29)
A	3	BM-MA MS, MM,
B	2	BM, MA, MS (2), NS, MM, EHR expert
C	4	BM, MM, MS, EHR expert
D	4	BM, MA, MS (3), NS
E	2	BM (is also MA), MS (5), EHR expert
External informants NS (2)		

*MA* Head of the Medical Administration, *MS* Medical specialist or resident, *NS* Nursing specialist, *MM* Medical manager, *BM* business manager

### Data collection

In selecting interviewees, we aimed to develop a comprehensive overview of the range of perspectives on collaboration. For each clinic, at least one medical specialist was interviewed. The administrative support perspective was included because the EHR introduction partly shifted administrative tasks from the medical administration to the medical professionals (medical specialists, nurse specialists, residents) so that registration could be realized at source. The managerial perspective was included because managers of the outpatient clinics had a keen interest in enhanced multidisciplinary collaboration.

Interviewees were selected with the support of two managers who had been involved in the EHR implementation. Initially, only those disciplines that had a clear role in the care delivery or fulfilled a management role were selected: heads of the medical administration, medical specialists, medical managers, and the business managers of outpatient clinics. Interviewees were invited by mail, and all invitees accepted the invitation. Once they had accepted the invitation, appointments were scheduled. Following a theoretical sampling logic [41], if the initial interviews showed that other disciplines were involved in a clinic’s collaborative practices, these other disciplines would be added to the list of interviewees (see Table 1). Similarly, three interviewees suggested to have an EHR expert present during the interview to help in explaining their experiences with the system, which resulted in three two-person interviews. Interviewees cautioned that the interview data might be negatively biased since the hospital was still in the post-implementation phase. To check this, two additional interviews were held (by phone) with two nurse specialists of a hospital that had already been working with the same EHR suite for five years. Thus, in total, 29 people were interviewed of which 27 were employed at the focal hospital.

Data were collected between September 2018 and February 2019. The face-to-face interviews at the clinic were all were voice-recorded with the exception of one interview where we took notes during the conversation. The interviews were semi-structured, leaving room for further probing and lasted 25–45 min. The interview protocol included questions on the four collaborative affordances proposed by Bardram and Houben [22]: (1) Portability, (2) Co-located access, (3) Shared overview, and (4) Mutual awareness (see Additional File 1). Moreover, open questions about the EHR’s effect on collaboration made it possible for further affordances to emerge.

### Data analysis

We analyzed the data by following the four steps.

- *Step 1.* All the interview data were transcribed and read through thoroughly for each outpatient clinic individually.
- *Step 2.* Codes were generated by two coders to create a comprehensive codebook (see Additional File 2). This data analysis approach was chosen because it allowed the context of each outpatient clinic to be kept in mind and offered opportunities to discover the facilitating or constraining conditions for collaborative affordance actualization.
- *Step 3.* Data were coded that could be associated with collaborative affordances, or collaboration within and between specialties, or collaboration within and between disciplines, or that concerned facilitating or constraining conditions for affordance actualization [42]. The coding procedure was primarily inductive within the four collaborative affordance categories deductively derived from the literature (Portability, Co-located access, Shared overview and Mutual awareness (see [14]), while maintaining an open eye for other affordances or themes (see codebook in Additional File 2. The inductive codes were derived from the data [43]. First, the emerging themes were described by using a first-order code that preserved the practitioner’s voice. Second, these first-order codes were classified into second-order code groups.
- *Step 4.* Second-order codes were aggregated into seven themes, consisting of the four initial collaborative affordances, two additional ones (Messaging and Orchestrating), and Conditions for affordance actualization. Finally, these aggregated themes and the included first-order and second-order codes were cross-checked among the cases.

## Results

This section reports on the cross-case analysis, with the underlying, within-case descriptions available in Additional File 3. Table 2 summarizes how the six identified collaborative affordances of the EHR facilitated and/or constrained collaboration within and between disciplines and medical specialties.

**Table 2:** Overview of facilitating and constraining influences of the EHR’s inscribed collaborative affordances

*Inscribed collaborative affordances:*	*Users recognized that the EHR****facilitates...***	*Users recognized that the EHR* ***constrains ...***
**Portability**	… accessibility of patient data, independent of location and medical context.	… digital sharing of patient data with health providers outside the hospital.
	… integration of patient data from different specialties, resulting in a comprehensive overview.	...mutual understanding of patient data because of specialty- and discipline-specific user-interfaces.
**Co-located access**	… professionals viewing the same data from different locations.	… modifying health records and entering orders simultaneously (by different professionals).
		… a comprehensive overview during multidisciplinary meetings because of a lack of desktops.
**Shared overview**	… integration and availability of patient information, avoids multiple data sources and handwritten notes.	… cognitively processing the overview. Information overload is experienced, due to the large number of notes and patient information not being presented in a chronological order.
	… once-only registration (at the source) and full registration of activities through orders.	… generating a cross-specialty overview since patient data are specialty- and department- specific. Departments and specialties use medical history and problem lists in different ways, leading to incomplete files.
**Mutual awareness**	… hospital-wide working processes.	… obtaining an easy-to-use overview due to information overload and patient information not being presented in a chronological order.
	… notification of results, quick updates.	… a shared awareness because patient data models are specialty- and department- specific.
**Messaging**	… discussing patients with other specialties without needing to refer them to the other specialties.	… face-to-face communication. The reduced need for face-to-face communication saves time but is experienced as reducing the collective responsibility for a smooth workflow.
	… the replacement of other messaging systems.	… an easy overview due to message overload.
	...uniform forms of communication.	
**Orchestrating**	… efficient and shared working processes.	… flexible task distribution. Strict authorizations constrain flexible, multidisciplinary task distribution.
	… the systematic registration of results.	… process efficiency due to a strict focus on orders.
		… ad hoc, diverse forms of collaboration. The EHR system enforces system-supported forms of collaboration. Some multidisciplinary consultations are not supported by the EHR.

### Affordance 1: portability

A widely shared view among the interviewed representatives of the clinics was that information from each specialty was integrated in the EHR. The former (legacy) system had already provided this functionality but the EHR ensured that notes by Medical Specialists were now also included. Several functional groups voiced the importance of collecting data from all specialties since this resulted in a comprehensive overview of the available information. Based on this shared overview, the medical specialists were better able to develop mutual awareness. This was most strongly expressed in the most intensively collaborating outpatient clinics (A, C, and D).

In three clinics (A, C, and E), the Medical Specialists argued that photographs imported into the EHR were sometimes still only accessible by certain clinical specialties. As a result, medical specialists were not able to discuss these images during meetings. This was experienced as a negative influence on collaboration. Moreover, it was argued that, in all cases, the health records of hospitalized patients were still tied to specific medical domains. As a consequence, medical specialists could only access these health records if they logged onto specific domains. However, no one commented that this negatively affected collaboration.

Interviewees from three clinics (B, D, and E) commented that data could not be shared with neighboring-hospitals through the EHR. Since these clinics receive many patients from other hospitals, this interfered with communication with medical specialists from the neighboring hospitals which hindered mutual awareness. Further, outpatient clinic B works intensively with external parties such as research institutions and its Business Manager indicated the difficulties in sharing relevant EHR data with these parties: *“I find it a big disadvantage that we cannot easily get reports from the EHR. We can’t do that ourselves: we are constantly dependent on others! [...]. But, we as [specific specialty] have to share lots of data with external agencies and we have struggled with that for a long time.”* - [B-BM1].

### Affordance 2: co-located access

In all the clinics, respondents mentioned that the EHR enabled simultaneous access to health records, but also complained that the EHR prevented users simultaneously modifying health records. Several medical specialists from all the outpatient clinics mentioned that they could not place orders when a colleague was working on the same health record.

It was also striking that the different functional groups who collaborated in the same office or clinic were frequently hindered. For example, three medical specialists and medical residents of outpatient clinic E mentioned that they, temporarily, could not complete their work during joint consultations with nursing specialists. In such situations, only one professional could have access to the health record. As a consequence, another professional was denied access and was therefore unable to process orders or relevant data in the EHR. Whether this should be an issue was questioned by one medical specialist since he was convinced that EHR users would often be working on different parts of the EHR database and could therefore not imagine that co-located access needed to be obstructed. Moreover, he argued that it would only make sense to impede co-located access when professionals were trying to work on the same part of the EHR database.

A very large number of notes were created in the EHR, in part because these could only be changed by their owners. Concerns were expressed in outpatient clinics A, C, D, and E about the quality of the shared overview since this was complicated by dozens of notes by various specialists. On a different but related issue, medical specialists were hindered in collaborating during multidisciplinary meetings when they had access to only one desktop because they then had to switch between medical results and the notes of the meeting which made it difficult to remember which patient was being discussed. Some had already seen that this could resolved by using a second desktop.

### Affordance 3: shared overview

As mentioned above, the large number of notes negatively affected the quality of the shared overview. As a result, medical specialists of outpatient clinics A, C, D, and E commented that they were hindered in gaining a mutual awareness of other specialists’ notes. Since data were ordered on priority and not on the chronology of events, all the interviewed medical specialists felt impeded in easily understanding what had occurred in the medical timeline of their patients. Interviewees from outpatient clinics B, D, and E commented that handwritten notes were something from the past, because notes were now entered in the EHR. Therefore, they argued, medical specialists should *“finally”* be able to understand the notes of their colleagues.

In all the outpatient clinics, medical specialists argued that the medical history and problem lists of patients were not useful in gaining a mutual awareness of the issues with other specialties. Two reasons emerged from the interviews. First, within specialties, there are different views on which information was important for providing high quality care. Therefore, within specialties, data were entered in different ways, resulting in specialty-specific information that was less useful for other specialties. Second, it was even argued that, within some specialties, no use was made of the medical history and problem lists, leading to friction between specialties when patients were referred with an empty health record when it came to certain specialties. Interestingly, some specialties involved in outpatient clinics A and C had developed a uniform policy for the use of the medical history and problem lists. As a result, all the medical specialists of these specialties entered the required data.

The importance of integrated information resources in providing high quality care was expressed by many medical specialists. For example, for some outpatient clinics (D and E), the medical history and problem lists were seen as highly important since these clinics often treat patients with an extensive medical history. However, as already mentioned, there were concerns about the quality of the data. Indeed, some interviewees of outpatient clinics D and E said that they did not make use of the medical history in their medical consultations and surgeries because they simply did not trust the data stored in the system. Specifically, some medical specialists and medical residents commented that important information was occasionally missing from the medical history and problem lists. Consequently, these interviewees explicitly read letters (as contained in the EHR) to develop a mutual awareness with other medical specialists.

Data included in the medical history and problem lists were tied to certain codes of the EHR’s vocabulary. As a result, symptoms that were not in its vocabulary, or symptoms that were misspelt could not be added. In this respect, the medical specialists of outpatient clinic E highlighted that the EHR did not include an adequate search functionality, impeding them in connecting the correct diagnosis with an appropriate code. This was considered to decrease the quality of the shared overview.

In all the clinics, it was argued that the provision of a shared overview is a requirement for collaboration between specialties as it increases their mutual awareness. However, this would only be effective if all the hospital’s specialties used the EHR consistently, which was not the case.

### Affordance 4: mutual awareness

In each of the clinics, interviewees argued that medical specialists were impeded in developing a mutual awareness between specialties because information was not clearly represented in the EHR. Two causes were offered: (1) the shared overview was not clear because each specialty entered the data differently, which negatively influenced the mutual awareness between medical specialists of different specialties, and (2) the data in the EHR were sorted on priority what impeded specialists in seeing what had happened in the medical timelines of their patients. As discussed earlier, the mutual awareness of some medical specialists from outpatient clinics A, C, and E was decreased because images were still tied to certain departments.

On the other hand, the EHR could support medical specialists in improving their *mutual awareness* of patients’ medical timelines since this process was now more transparent. Moreover, the *mutual awareness* between medical specialists was increased due to portable notes. With all the specialties of the hospital integrated in the EHR, interviewees in four outpatient clinics (B, C, D, and E) commented that patients could be referred more easily between different specialties by means of the *orchestrating* affordance. The use of the *messaging* affordance was also seen as an important component in supporting mutual awareness between medical specialists.

No single functionality of the EHR could be directly linked to the mutual awareness of healthcare professionals. However, all the other collaborative affordances had an influence on actors’ mutual awareness. Therefore, the mutual awareness between different healthcare professionals was seen by many interviewees as a highly important factor in collaboration. However, the mutual awareness between medical specialists depended on uniform use of the EHR.

### Affordance 5: messaging

The advantages of the messaging affordance were experienced differently in each outpatient clinic. However, interviewees in all the clinics appreciated the benefit of having the possibility to attach health records to messages. Previously, patient-related matters were discussed through Outlook. This frequently led to misunderstandings between medical specialists because health records could not be attached to an email. In three outpatient clinics (A, C, and D), it was mentioned that some specialties had developed a policy that required the use the messaging option. Through this, various functional groups within these specialties could be assured that the “receiver” had actually read their message. Accordingly, the healthcare professionals in these specialties were better equipped to gain a mutual awareness.

Conversely, in clinics B and E, there was no observed shift to adopt messaging. Specialties in these outpatient clinics did not adopt a uniform policy for the use of the messaging affordance. In these cases, the collaborative advantages depended on the medical specialists’ individual decisions to use the afforded messaging. Certainly, some of the medical specialists in outpatient clinic E did not use messaging. Interviewees from both clinics B and E commented that some medical administrators did use messaging, but that some medical specialists did not. As a result, the medical administrators’ messages were not answered.

As such, the delivery of the collaborative advantages offered by the messaging affordance depended on its uniform use in and between clinics. It was widely argued that the messaging opportunity currently led to an information overload, mostly due to it being used for uninformative medical results or letters. A decrease in the use of the messaging option had been noted in outpatient clinics B and E. Side effects of the messaging system were also expressed. For example, verbal communication between collaborating disciplines was lost in most cases, and interviewees saw this as negatively impacting on their collaboration.

### Affordance 6: orchestrating

All the business managers and medical administrators argued that the use of the orchestrating affordance was more efficient than the use of paper notes because orders were processed immediately and sent to the correct actor. On the other hand, most medical specialists argued that the use of the orchestrating affordance led to time consuming digitalized healthcare processes and marginalized verbal communication with colleagues, patients and the medical administration. As reported by previous studies [17, 18], the time spent to use the EHR was experienced to decrease the time spent on verbal communication with colleagues and patients. Here, this was seen to negatively affect the collaboration between different functional groups and the quality of healthcare. This was expressed by a Medical specialist as follows: *“The disadvantage is that the EHR takes away the interaction between people and I think that is actually a drawback: interactions between people are often more useful than the parametric recording of data. We have to make sure that we don’t diminish this interaction too much.”* - [D-MS3]. Hence, making use of the orchestrating affordance was seen as boosting efficiency from a managerial perspective but was perceived as less desirable from a patient care standpoint.

In four outpatient clinics (B, C, D, and E), it was voiced that different disciplines were not able to work in a natural way because all processes were now based on digitalized orders. Examples were provided of some disciplines not collaborating without an order as this was the hospital’s policy. For example, daily face-to-face collaborative processes between nurses and medical specialists were hindered by this in outpatient clinics B, D, and E.

Most interviewees argued that the orchestrating affordance did not easily support them in arranging multidisciplinary consultations with other specialties. Although the nature of these consultations was not affected, the orchestrating opportunity did not properly guide financial information flows within the hospital. As a result, some outpatient clinics did not receive financial compensation for organizing these meetings. As a consequence, one of outpatient clinic A’s specialties was in debt for organizing these consultations. Moreover, medical specialists from outpatient clinics D and E mentioned that it was too difficult to arrange multidisciplinary consultations through the EHR. Consequently, these interviewees did not make use of this functionality.

Some medical specialists raised the difficulty of inviting other medical specialists to consultations. One medical specialist argued that this could be seen more as a future potential of the EHR to further support the collaboration between different specialties. However, several business managers and medical specialists expressed the view that multidisciplinary meetings were better registered in the EHR and that the results of these meetings could more easily be found than before.

#### Influence of organizational choices and policies

EHR users interviewed from all the clinics agreed that the strict role authorization and different system representations in the EHR hindered interdisciplinary collaboration. The hospital had only authorized medical specialists to enter patient-related data in the EHR. These strict role authorizations limited interdisciplinary collaboration as a business manager explained: “*The Board of our organization decided that only medical specialists would be authorized to enter patient-related data. Therefore, the medical administrators are no longer authorized although it is, of course, a team that is collaborating*.” [C-BM1]. Before the introduction of the EHR, medical administrators were authorized to enter patient-related data and, therefore, a shift was perceived in the administrative burden from the medical administration to the medical specialists.

Various medical specialties found that they were not able to collaborate well because each specialty worked in a different medical context in the EHR. As a result, EHR users were hindered in understanding what had taken place when something went wrong in terms of orchestrating because the two parties had different system representations.

In two outpatient clinics (A and D), the lack of organization-wide policies made it difficult to actualize the collaborative affordances. However, all the medical specialties involved were required to use the EHR according to the department’s own policy. As such, all the specialties were assured that certain affordances were used. As a result, actors were guaranteed that their messages would be actually read by the right actor. In the other outpatient clinics (B, C, and E), no indications were found that a department-wide policy had been implemented. As a consequence, collaborative affordances were perceived and actualized differently by the various medical specialists.

## Discussion

The core processes seen in hospitals are highly collaborative in nature and many hospitals have implemented comprehensive Electronic Health Records to facilitate multidisciplinary collaboration. By adopting an affordance lens, this study has examined how an Electronic Health Record facilitates or impedes collaboration in five outpatient clinics. Through an analysis of the interplay between collaborative EHR affordances, we contribute to the literature by demonstrating how multidisciplinary collaboration is not only facilitated but also constrained, and how EHRs can have intended as well as unintended impacts on collaboration [9].

This study draws on Bardram and Houben’s [22] identification of four collaborative affordances (Portability, Co-located access, Shared overview and Mutual awareness) and complements their findings with two additional, inductively derived, collaborative affordances: Messaging and Orchestrating. The labelling and definitions of the latter two were aligned with the research of Chase [11]. Messaging concerns information transfer and communication between professionals, and also with other members of the hospital. Orchestrating ensures that the right person is doing the right thing at the right time for the patient. This study illustrates that the introduction of an EHR changes important structuring devices in the organizational processes of hospitals [44].

Our study shows that Electronic Health Records facilitate collaboration through the systematic integration of patient data from different specialties, which results in a shared and comprehensive health record to which users can have simultaneous access independent of time and place. This promotes mutual understanding and enables health professionals to coordinate their activities and prevents the duplication of activities such as tests. At the focal hospital, the EHR served as a joint communication channel. This discouraged health professionals from continuing with their handwritten notes and their local health records, and promoted the use of shared data. The EHR also facilitates collaboration by providing the information necessary for joint clinical decision-making, which is especially important for the quality of care of chronic patients who are often treated by several medical specialists. The EHR also promotes hospital-wide shared working processes, which creates conditions necessary for collaboration.

However, our study also shows how the EHR in the focal hospital can at times inhibit multidisciplinary collaboration [10–13]. Many specialty- and discipline-specific user interfaces were seen as constraining a mutual understanding of patient data. Medical departments utilized particular functionalities, such as medical histories and problem lists, in different ways, making the EHR less reliable and thereby complicating collaboration. Many physicians also argued that entering and reading large amounts of patient information is time consuming, creates information overload and harms effective collaboration. This was partly caused by the specific use of the system, e.g. physicians posting numerous individual notes. The EHR reduces the need for direct face-to-face communication which is intended to save time, but is experienced as hindering a smooth workflow and the building of mutual trust and effective collaboration. Several of the interviewed medical specialists expressed the risks they associated with receiving electronic warnings of life-threatening medical results by means of the messaging function. Before the introduction of the EHR, medical specialists were informed of such results by phone or face-to-face. Finally, this EHR, with its inward-looking focus on the hospital, was seen as complicating smooth information sharing and collaboration with healthcare providers outside the hospital.

This study showed that shared data can be difficult to use when the user interfaces differ among work units. Leonardi [34] and Orlikowski [45] both demonstrated that the extent that the intended benefits of a new technology are reaped is dependent on how actors actualize its affordances. In the hospital studied, the clinics actualized the EHR collaborative affordances differently, resulting in constrained collaboration among medical specialties [45]. This implies that the enforcement of hospital-wide policies on the use of EHRs are necessary to reap the potential benefits of these systems. This study also supports the research of Thapa and Sein [40] who argue that contextual factors largely determine the extent to which collaborative affordances are actualized by groups of individuals. Our findings show that EHR-enabled collaboration is dependent on contextual factors including role authorizations, system representations, organizational policies, and how medical professionals use the collaborative affordances.

### Practical implications

This study shows that both healthcare organizations and EHR providers should be aware of several issues related to the collaborative affordances of EHRs. First, hospitals consist of clinical departments that have different working routines and expectations from an EHR [22]. This implies that policies are needed to achieve effective collaboration among departments through an EHR. Without organization-wide policies, departments may actualize collaborative affordances differently and, as a result, as this study showed, the mutual awareness and common ground between different healthcare professionals can be harmed. One of these policies is to involve the different medical disciplines and to achieve shared decision-making and ownership regarding the selection, implementation and adaptation of collaborative technologies, such as EHRs. Hospitals should also recognize the downside of strict role-authorizations in EHRs as our findings indicate that these negatively influenced collaboration among different functional groups. Further, practitioners need to be aware of the drawbacks of the different system-representations (medical contexts) in EHRs and how these may negatively affect collaboration on the clinical department level. Finally, this study shows that EHRs can both support and impede collaboration on different organizational levels. Since achieving the advantages is dependent on the affordance actualization process adopted by multiple clinical departments and disciplines, managers should carefully guide this process if they wish to reap the full potential benefits in terms of an EHR’s collaborative affordances.

### Limitations and opportunities for further research

This study focused on five outpatient clinics that all involved multiple specialties. We recognize that including collaborative practices in and with other clinical facilities as well as interorganizational collaboration might have resulted in additional perspectives on how each of the EHR’s affordances facilitate or constrain collaboration. We also acknowledge that this study was conducted at only one institution with one EHR system. Previous research [46] shows wide variation in how different institutions use the same EHR product in different ways and how different EHR products offer different functionalities. Another limitation is that this EHR system was implemented only one year before we conducted this study. We can imagine that this relatively short timeframe has influenced the study’s outcome. When users become more familiar with the affordances, they may find converging ways to make it instrumental per type of multi-disciplinary collaboration. Therefore, we would urge further research on how hospitals and EHR providers can overcome the constraining influences we identified, notably those related to each discipline having different system representations. We saw that this discipline-related system representation afforded efficiency in electronic record use within a discipline and prevented information overload, but hindered the cross- and interdisciplinary collaboration needed for integrated patient care. Another relevant direction for further research is to examine how the collaborative affordances of an EHR impact the care provider – patient relationship and the resulting quality of clinical care.

## Conclusions

The aim of this study was to examine how the collaborative affordances of an EHR are actualized in its use by the disciplines responsible for patient care in outpatient clinics. Our findings indicate that the EHR’s affordances do have the intended facilitating influences on collaboration but, simultaneously and unintendedly, constrain collaboration in other ways. This prevented full actualization of the collaborative affordances in the focal hospital. In order to actualize the collaborative affordances of EHRs more fully, health professionals need to be able to retrieve, understand, and trust each other’s information. Only then can they rely on each other’s appropriate and timely use of the system. Such multifaceted trust can gradually develop through hospital-wide policies that stimulate a more coordinated use of the system, which may involve the formal recognition of positive workarounds to counter the constraints identified. Further research is needed to determine which organizational, technical, and behavioral adaptations can more fundamentally solve the constraining influences.

## Supplementary information

**Additional file 1.** Interview protocol.

**Additional file 2.** Codebook.

**Additional file 3.** Case descriptions of the outpatient clinics (A to E).

## Data Availability

The datasets used and/or analyzed during the current study are available from the corresponding author on reasonable request.

## Abbreviations

EHR: Electronic Health Record
MA: Head of the Medical Administration
MS: Medical specialist or resident
NS: Nursing specialist
MM: Medical manager
BM: Business manager
