# Supplementary material for: The influence of electronic health record use on collaboration among medical specialties
Source: BMC Health Serv Res. 2020 Jul 22;20:676. doi: 10.1186/s12913-020-05542-6 (PMC7374868; doi:10.1186/s12913-020-05542-6)
Supplement: Supplementary file 1 — Additional file 1. Interview protocol. [file 12913_2020_5542_MOESM1_ESM.docx]

**Additional File 1. Interview protocol**

**Impact of the EHR on working processes and affordances**

1. How do you use the EHR to carry out your daily activities?

*Probe 1:* *If you compare this with the situation before implementation, how is this different?*

1. What do you consider the most important advantages and disadvantages of the HER?
2. How does the EHR influence the time required for performing working processes?

*Probe 1: Could you give an example?*

*Probe 2: How does this affect patients’ trajectories according to you?*

*Probe 3:Do you have the feeling that u need more or less time per patient due to working with the EHR?*

1. How do system functionalities influence your working processes within the EHR?

*Probe 1: In what way? Examples?*

*Probe 2: Which functionalities are missing but needed according to you? Why?*

1. How does the EHR influence standardization of working processes?

*Probe 1: How does this affect cooperation and referrals of patients between the different specialties in your clinic?*

*Probe 3: How does standardization affect patients’ trajectories according to you? Example?*

1. How does the EHR influence safety and accessibility of data? You can also think about information reliability, information consistency and privacy of patients.

*Probe 1: In what way? Examples?*

**Collaboration**

1. To what degree do medical specialties of this outpatient clinic collaborate with each other?
2. What system functionalities provide opportunities to collaborate?

*Probe 1: To what degree do you make use of these functionalities?*

*Probe 2: How do these functionalities influence collaboration with your colleagues (nurses, administrators)?*

*Probe 3: How do these functionalities influence collaboration with other medical-specialties of this clinic?*

*Probe 4: How do these functionalities influence collaboration with other medical-specialisms (outside clinic)?*

*Probe 5: Are you currently satisfied with how collaboration takes place with other medical specialties? Why?*

1. How does this EHR provide opportunities to open and/or bring health records at (to) different locations in this hospital? (Portability)

*Probe 1:How does this, in your opinion, influence collaboration with other medical specialties? How would you describe this for your Clinic?*

1. To what extent are you able to view patient files simultaneously (in the same room) with your colleagues? (Collocated access)

*Probe 1: How does this influence collaboration with other disciplines?*

*Probe 2: How does this influence collaboration with other medical specialties?*

1. How does the EHR offer possibilities to work on a shared health record with other colleagues? (Shared overview)

*Probe 1: How does this influence collaboration with other disciplines?*

*Probe 2: How does this influence collaboration with other medical specialties?*

1. To what extent does the EHR offer opportunities to keep track of the medical development of patients together with other specialties? (Mutual awareness)

*Probe 1: How does this influence collaboration between different specialties?*

1. Are there, in your opinion, any other ways how the EHR influences collaboration between different medical-specialisms?

*Probe 1: What influence does this have on the collaboration within the outpatient clinic?*

*Probe 2: Does this facilitate or constrain collaboration between different disciplines?*

1. In what way could the EHR be adapted to (further) facilitate collaboration?
