# Supplementary material for: The influence of electronic health record use on collaboration among medical specialties
Source: BMC Health Serv Res. 2020 Jul 22;20:676. doi: 10.1186/s12913-020-05542-6 (PMC7374868; doi:10.1186/s12913-020-05542-6)
Supplement: Supplementary file 2 — Additional file 2. Codebook. [file 12913_2020_5542_MOESM2_ESM.docx]

**Additional File 2: Codebook**

| **Collaborative affordance** | **Descriptive code, per affordance** | **Representative Quotation** |
| --- | --- | --- |
| **Portability** | | |
| *Facilitating* | Accessibility of patient data, independent of location and medical context. | "[…] anyway, everything is in one file. [...]. That is really different from the past: if we received a patient from [names specialty] then we were not able to find any information of the patient. It was not possible to look into their files." - [D-MS2] |
|  | Integration of patient data from different specialties. | “I hear from medical specialists that they’re able to see what other consultations are planned, the process is more transparent.” – [B-BM1] |
| *Constraining* | Digital sharing of patient data with health providers outside the hospital. | "They have a lot of information in their system, but what does this colleague [from other hospital] do? He prints the health record, sends it by fax, we scan it, and then we have a scanned letter in the EHR. And then we talk about two hospitals with the same EHR. Come on… " - [D-MS3] |
|  | Mutual understanding of patient data because of specialty- and discipline-specific user-interfaces. | "The emergency-department acts as a single hospital. When I want to search patients in the EHR that are located in the emergency-department, then I first have log in to the emergency-domain to get the right authorizations to log in." - [C-MM1] |
| **Co-located access** | | |
| *Facilitating* | Professionals viewing the same data from different locations. | “When I go to Surgery, I can sit there behind a computer and look into a patient file together with my colleagues […]. Also from other locations you can use your account to open patient files.” [D-NS1] |
| *Constraining* | Modifying health records and entering orders simultaneously (by different professionals). | "If we are together with other specialists then we are not able to write in each other's notes. We sometimes run into that. As a result, you always have to create a new note. After a while you get hundreds of notes [..]. That’s unstructured and unclear." - [A-MS2] |
|  | A comprehensive overview during multidisciplinary meetings because of a lack of desktops. | “Within the [specific specialty’s] meeting we often click through to different screens, such as [specific specialty], which you should be able to do in one screen." - [D-MS1] |
| **Shared overview** | | |
| *Facilitating* | Integration and availability of patient information, avoids multiple data sources and hand written notes. | “In terms of the outpatient clinic the system works fine: you work with one system, for instance, the Cardiologist, who also uses the same medication unit now, where previously personal notes were kept, that is now all just in one file, you see what he or she has written down.” – [D-MS2] |
|  | Once-only registration (only) at the source and full registration of activities through orders. | "At the moment we have the following question: What is registration at the source [enter relevant patient-data in the EHR]? Should that really be the medical specialist who does that or can it also be the medical administrator? The Board have clearly said: 'Only doctors have the qualifications to register, but we know better than anyone else that doctors don’t always have the right administrative understanding. [...]. I think clear boundaries have not yet been determined.” – [E-MA1] |
| *Constraining* | Cognitively processing the overview. (Information overload is experienced, due to a large number of notes and patient information not being presented in a chronological order.) | "You have to search for the information. The information overview is very unclear, but at a certain point you will probably learn to look through it. I personally like it when information-transfer goes fast and when information is easy to understand." - [A-MS1] |
|  | Generating a cross-specialty overview since patient data are specialty- and department-specific. Departments and specialties use medical history and problem lists in different ways, leading to incomplete files. | "The medical history is as good as it is entered. However, at the moment, the Medical history is more like a broad history. For example, when I see a patient who has had a scar surgery twice before, then I want to know in detail what happened and what went wrong. It could be in the system, but most of the time it is not. - [E-MS2] |
| **Mutual awareness** | | |
| *Facilitating* | Hospital-wide processes (uniform working processes) | "I also hear from medical specialists that they're able to see what other consultations are arranged, the process is more transparent." - [B-BM1] |
|  | Notification of results, quick updates | “What I find very important is that we get reports of new results. Therefore, I am better able to see the results of [specific department] and of [specific department]. Where I previously had to go to the clinic to check whether I already had received results – and where you even could overlook these results - are these results now sent to me personally.” - [C-MS1]. |
| *Constraining* | Obtaining an easy-to-use overview, due to information overload and patient information not being presented in a chronological order. | "It is possible to maintain an awareness of the progress of a disease, but it is less efficient than the situation before. […] we have not discussed and arranged it properly: a problem list is not ordered chronologically at the moment, but on the basis of the type of problem." [...] I do not see the sequence of events, while that is extremely important." - [D-MS3] |
|  | Shared awareness because patient data models are specialty- and department-specific (no uniform use). | “The concept of a shared history is good. [..]. But it is a bit strange, because an Internist, a Urologist or a Surgeon all have different perspectives on how you should document symptoms and what you should document: [gives an example]. The ambition of uniformity is of course great, but I think that each medical specialty have different views of the Medical history." - [E-MS3] |
| **Messaging** | | |
| *Facilitating* | To discuss patients with other specialties without needing them to refer them to the other specialties. | "That is a nice development. We also agreed that we will use the Messaging as the exclusive means to discuss patient-related affairs. That works really well.” - [A-MM1]. |
|  | The replacement of other messaging systems (enabled transition to the EHR). | “A lot of messages enter through Messaging. In fact, it has taken over the e-mail for the large part, which has enabled the transition towards the EHR” – [C-MM1] |
|  | Uniform forms of communication. | "The EHR also has become a communication channel because we also receive messages. With these messages you'll be updated immediately about results or letters that were composed incorrectly [etc.]." - [E-MS1] |
| *Constraining* | Face-to-face communication. The reduced need for face-to-face communication saves time, but is experienced as reducing the collective responsibility for a smooth workflow. | “Verbal communication between people is reduced. So, you have to secure many more things in work processes in order to run processes smoothly.” - [C-MM1] |
|  | An easy overview due to message overload. | “…he [mentions a specific medical specialist] received over 250 messages a day.” – [B-BM1]  "All kinds of messages are to be found in the Messenger: results of patients, [..], letters that you have to correct, but also just emails from colleagues and secretaries. […]. It is good that we have a communication channel like that, but in practice, it is really dramatic.” - [E-MS1] |
| **Orchestrating** | | |
| *Facilitating* | Efficient and shared working processes. | "Well, in the past medical specialists wrote the orders on a piece of paper, brought it to the secretary, and then the secretary processed the orders. Currently, medical specialists select the right order and the medical administration receives it on their worklist. It has become more efficient."- [B-BM1] |
|  | Systematic registration of results. | "Now the [specific specialty] can see the patients’ history. So, we only have to mention important [new/additional] information, instead of copying an entire list [...]. We can therefore ask more specific questions that are more relevant” - [C-MS1] |
| *Constraining* | A flexible task distribution. Strict authorizations constrain flexible, multidisciplinary task distribution. | "The very strict role-authorizations in the EHR [...] make the practical work difficult, because one role has certain things that the other role can’t see. Therefore, now we are not able to work in a more natural way with these strict role authorizations." - [B-MS2] |
|  | Process efficiency due to a strict focus on orders. | Currently, it sometimes happens that when a medical specialist says: "please take out the catheter" that nurses wait until they have received the order [through the system], well, that is of course not workable." - [D-MS2] |
|  | Ad hoc, diverse forms of collaboration. The EHR system enforces system-supported forms of collaboration. Some multidisciplinary consultations are not supported by the EHR. | "The downside of the system is that the medical specialists and the medical administrators have different system representations. Therefore, we are less aware of each other’s work during our collaboration. [...]. Sometimes you want to help another disciplinary group, but they have completely different system representations. That's a disadvantage" – [B-BM1] |
