# Supplementary material for: The influence of electronic health record use on collaboration among medical specialties
Source: BMC Health Serv Res. 2020 Jul 22;20:676. doi: 10.1186/s12913-020-05542-6 (PMC7374868; doi:10.1186/s12913-020-05542-6)
Supplement: Supplementary file 3 — Additional file 3. Case descriptions of the outpatient clinics (A to E). [file 12913_2020_5542_MOESM3_ESM.docx]

## **Additional File 3: Within case analyses: outpatient clinics A to E**

### Outpatient clinic A

#### Description of the outpatient clinic

Outpatient clinic A involves three specialties with some medical overlap. Further, many patients are referred by other specialties of the hospital and treated in this clinic. Although the three specialties have some medical overlap, they do not need direct collaboration to provide healthcare. Nevertheless, many multidisciplinary consultations are arranged in this outpatient clinic because most of the patients have clinically complex issues. However, all these multidisciplinary consultations are provided by the three specialties separately.

#### EHR’s facilitating influences on collaboration

The **mutual awareness** between the three specialties was strongly supported because all the disciplines use two specific collaborative affordances of the EHR: **orchestrating** and **messaging.** Through using these collaborative affordances, the medical specialists felt better supported in their collaboration since it had been agreed that these affordances should be used to organize patient-related affairs. As argued by the medical manager: *"That is a good development. We also agreed that we use the messaging affordance as the exclusive means to discuss patient-related affairs. That works really well.”* - [A-MM1].

However, although work-related processes had become more efficient, the adoption of **orchestrating** and **messaging** had limited oral communication between the various functional disciplines, as noted by the business manager: “*I think there is less oral communication. Also, because we now have the messaging facility, they* [the medical administrators] *see the doctors less in person."* - [A-BM1].

Every interviewee voiced that the EHR afforded **portability**. The interviewees felt supported in accessing health records at different locations by means of a desktop. A medical specialist mentioned that the nature of collaboration between the different specialties might change when the EHR could be accessed with other devices such as tablets and smartphones. All the interviewees argued that the data (such as notes and letters) of each specialty could be found in the **shared overview**. As a consequence, medical specialists felt better supported in collaborating with other specialties since it increased their **mutual awareness**. Finally, the EHR increased the **mutual awareness** of the different specialties because decisions could be based on more comprehensive information, as stated by a medical specialist: “*There are various specialties that provide information, such as lab results and x-ray images, that is now all integrated into the EHR, which is therefore positive"* - [A-MS1].

#### EHR’s constraining influences on collaboration

A number of interviewees commented that because the data of different specialties were sorted on priority and not on the chronology of medical interventions, the medical specialists were hindered in efficiently achieving **mutual awareness** of other specialties’ involvement in a patient’s treatment. Some interviewees complained that the EHR restricted medical specialists in importing images. Accordingly, this constrained them in being able to quickly discuss scans and images during a meeting.

Further, two interviewees argued that the limitations on the EHR affordance of **co-located access** to update a single health record constrained them in collaborating efficiently. Medical specialists were hindered in simultaneously working on a single note during meetings. This negatively affected them in attempts to build a clear **shared overview**. As a consequence, the **mutual awareness** between different medical specialists was lowered, as argued by a medical manager: *"We are not able to write in each other's notes when we are together with different specialists. We sometimes run into that, and it’s really annoying. You always have to create a new note. In the end, you get hundreds of notes*. [...]. *That is unstructured and unclear."* - [A-MM1]

### Outpatient clinic B

#### Description of the outpatient clinic

Outpatient clinic B consists of two distinct specialties that have no medical interfaces and consequently do not collaborate. As explained by a medical specialist: *"We are placed in the* [name of clinic] *clinic which sounds very integrated, but actually we have our work and they have their work"* - [B-MS2]*.* In other words, both specialties have different and separate medical treatment paths for their patients but are incorporated in the same outpatient clinic and, consequently, no advantages for multidisciplinary collaboration are experienced. Both specialties do however frequently get patient-related consultations from other specialties of the hospital.

#### EHR’s facilitating influences on collaboration

The healthcare professionals felt supported in their collaboration by having **portable** health records, because information from other specialties was not bound to a certain physical location. For example, previously, medical specialists had to first open a mailbox to see the results from other departments. These results can now be found in the **messaging** function. Therefore, as argued by the business manager, the **portability**, the **shared overview**, and the **orchestrating** affordances directly increased the **mutual** **awareness** between different specialties: *"I hear from nursing specialists that it is much easier to request* [e.g. by an order] *a consultation at* [specific specialty] *because we now have an overall picture. Previously, everybody had their own notes and every department its own way of working. Now, everything is located in one system and this helps when patients are referred between different specialties”-* [B-BM1].

A medical specialist mentioned that he appreciated the **messaging** option because questions were tied to patients’ health records. As a result, this medical specialist was better supported when providing answers to other specialties (increased **mutual awareness** between medical specialists from different specialties). The **mutual awareness** between medical specialists was also increased through the use of digital notes. The medical manager commented that he was finally able to understand the notes of his colleagues because these notes were no longer handwritten. Finally, the business manager argued that the use of the **orchestrating** affordance positively influenced the efficiency of collaboration: *"Well, in the past, physicians wrote orders on a piece of paper, brought it to the secretary, and then the secretary processed the orders. Currently, physicians just select the required order(s) and the administrator gets the order(s) immediately on the worklist. It has become more efficient”* – [B-BM1].

#### EHR’s constraining influences on collaboration

It became clear that the use of the **orchestrating** and **messaging** opportunities led to a decreased level of verbal communication within and between disciplines, which was seen as a negative development by all the interviewees. The business manager also explained that this outpatient clinic did not have a uniform policy on the use of **messaging** and that this occasionally hindered collaborating teams involving different disciplines: *"Well, sometimes we have irritations between the medical administrators and the medical specialists. For example, when an administrator sends a message to a doctor to call a patient. Well, that is difficult because the messaging system is sometimes not being used by the other side.”* - [B-BM1].

Concerns were expressed by all the interviewed medical specialists that **messaging** currently led to an information overload because different types of results (such as questions from the medical administration or from other medical specialists and laboratory results) were received through **messaging**. The medical manager of the clinic mentioned that *“…he received over 250 messages a day*.*”* As a consequence, some medical specialists had stopped using **messaging**. The business manager expressed the view that such non-use of **messaging** would lead to decreased **mutual** **awareness** between specialties: *“The majority of the doctors to some extent neglect* ***messaging****. However,* ***messaging*** *is also used to refer patients between specialties. I don’t think that all doctors are aware of all the messages and have a clear picture of their patient.. I think that things occasionally disappear.”* – [B-BM1].

Some interviewees stated that **co-located** **access** was inhibited by the EHR. Medical specialists felt hindered in simultaneously preparing consultations with nursing specialists. Although the **portability** should facilitate medical specialists in sharing health records across different locations, a medical specialist in this clinic explained that he was obstructed from digitally sharing health records with neighboring hospitals: *“It is really frustrating when a patient is referred from* [Hospital x]. *Then I have to process a scanned paper in our EHR but, sometimes, I am not able to find the information that was originally in the health record of* [Hospital x].*" -* [B-MS2].

### Outpatient clinic C

#### Description of the outpatient clinic

This clinic includes four specialties with no medical interfaces. Most patients have chronic diseases and therefore have an extensive medical history. One of the clinic’s specialties collaborates with many other specialties outside the clinic.

#### EHR’s facilitating influences on collaboration

This clinic’s patients are frequently referred to other specialties during their medical treatments. The EHR supports this process because the schedules of all specialties within the hospital have been integrated through the **orchestrating** affordance. Further, the interviewees expressed advantages of the use of **messaging**. For example, a medical specialist argued that the **mutual awareness** between specialties was increased by using **messaging** because a medical specialist would get notified by the EHR if there were new results: *“What I find very important is that we get reports of new results. Therefore, I am better able to see the results of* [specific department] *and of* [specific department]. *Where I previously had to go to the clinic to check whether I had already received results – and where you might even overlook these results - these results are now sent to me personally.”* - [C-MS1].

Second, the **mutual awareness** of medical specialists was increased because all the documents from every specialty were now integrated in the EHR. As a result, medical specialists felt better able to understand what had happened in the medical timeline of their patients because other specialties’ notes were now also integrated. The use of the **messaging**, **orchestrating**, and **shared overview** opportunities were generally seen as supportive of collaboration between specialties. For example, a medical specialist noted*:* *"Now the* [names a specific discipline] *can see the patient’s history, so we only have to mention important information instead of copying an entire list* [..]*. We can therefore ask specific questions that are more relevant.”* - [C-MS1]

#### EHR’s constraining influences on collaboration

First, several of the interviewees argued that **messaging** currently led to an information overload for medical specialists as they received so many messages. Second, **orchestrating** hindered collaboration among professionals when medical specialists forgot to process a required order. The business manager explained that everyday collaborative working processes were sometimes obstructed: “*We have had situations where nurses could not take blood samples because the order was missing. In such situations, the nurse had to call the medical specialist to ask if they could process the appropriate order. In the meantime, the patient had to wait another 15 minutes.”* – [C-BM1]

One medical specialist claimed that he was impeded in opening health records of hospitalized patients when he was working within a certain medical domain. To circumvent this problem, the interviewee had to log in to a different medical domain in order to access the health record. This indicates that health records were still sometimes bound to a certain medical context and thus not always fully **portable**.

Some medical specialists also expressed a difficulty in developing an understanding of a patient’s treatment path because notes were not clearly represented in the **shared overview**. Consequently, the medical specialists felt hindered in efficiently developing a **mutual** **awareness** of what other specialties had done earlier, as illustrated by a medical specialist*: "One of the key drawbacks of the notes is that everyone works in the same field. We cannot distinguish the important treatment advice from less important ones. Also, there is no distinction between notes from outpatient clinics and notes related to hospitalized patients. It is all mixed up."* - [C-MS1]

As in other clinics, **co-located access** to health records was impeded by the EHR. As a result, two medical specialists felt that they were hindered in proceeding with their work when a colleague was working on the same health record. It was also voiced that the EHR led to a decrease in verbal communication, as emphasized by a medical specialist*: “Verbal communication between people does not exist. So, you now have to secure many more things in work processes in order to run processes smoothly.”* - [C-MS2]

In this clinic, it was specifically expressed that the EHR did not support multidisciplinary meetings as it could not provide all the information needed for these meetings. As a result, medical specialists felt unable to efficiently create a **mutual awareness** of a patient’s situation. Therefore, one specialty decided to retain their legacy system in order to support their multidisciplinary meetings: *"At the moment, the EHR is not well equipped to visualize and present a good overview of the entire* [mentions name of a specialty] *follow-up. We simply have too little overview of a patient’s state of affairs. Consequently, we have insufficient information to support the multidisciplinary meeting.”* – [C-MS1].

### Outpatient clinic D

#### Description of the outpatient clinic

Clinic D involves four specialties. Further, it has a separate department where medical specialists from various specialties collaborate intensively to treat patients that have a long medical history. Consequently, the EHR is an important tool for these medical specialists to be able to understand the medical timelines of their patients. The business manager explained that the four incorporated specialties of this clinic hardly collaborate: “*We have* [mentions the three specialties: A, B, and C]*. Between* [A] *and* [B]*, there is hardly any collaboration as these specialties do not have any medical interfaces. C has more collaborative relationships, including with* [mentions another specialty]*, but this specialty is not incorporated in our clinic”* - [D-BM1]*.*

#### EHR’s facilitating influences on collaboration

First, because health records are **portable** in the EHR, medical specialists now felt better equipped to have meetings with other specialties and with neighboring hospitals. Second, collaboration between specialties was better supported since data from all the hospital’s specialties were integrated in the EHR, as highlighted by the medical manager: *“We all look at the same screen and go through the same processes.* [...]*. Previously, many specialties had different types of files that no one else could access.”* - [D-MM1] Furthermore, the EHR enabled medical specialists to see who had changed a file, who else had approached a specific patient, and if any appointments were scheduled with other specialties. As a result, medical specialists felt they were better supported in gaining a **mutual awareness**, together with other specialties’ physicians, of the medical timeline of their patients.

Second, the interviewed medical specialists argued that their **mutual awareness** with nursing specialists had increased because both disciplines no longer used *“extensive”* paper notes. The information contained could now be easily obtained from the EHR because the displayed information was clear and discrete.

#### EHR’s constraining influences on collaboration

The EHR hindered collaboration during meetings because it could not provide a clear **shared overview**. Medical specialists had to search for the required information during multidisciplinary meetings with other medical specialists and this hindered their collaboration: “*What currently happens at our meeting is that we constantly switch between the different scans,* [specific] *results and that kind of thing* [...]*. This has an influence on our working processes because, when you look at those (shifting) results, you can forget which patient is being discussed.”* - [D-MS1]. The same interviewee suggested that this problem could be solved by using two desktops. In this way, there would be no need to switch between different notes and medical results, which would positively influence **mutual awareness**.

Second, all the disciplinary groups considered **co-located access** to place orders for a specific patient or to update a single health record was obstructed. A medical specialist explained that he and his collaborating colleague used different parts of the EHR system. For him, it was very difficult to then understand why the hospital had chosen to block all disciplines from having simultaneous access to the same health record. Given that data were sorted on priority and not on the chronology of events, the **shared overview** indirectly hindered medical specialists in efficiently gaining a **mutual awareness** with other specialties: *“It is all in one system, but it is still specialty-specific*. [...]. *Further, it sorts on priority and not on chronology. Doctors want to see the chronology of events. The way it is sorted now, complicates working with multiple specialties."* - [D-MS1]

Although the hospital had some policies in place prescribing how specific functionalities should be used, many specialties did not follow them. Consequently, data was entered differently by each specialty and this negatively affected the quality of the **shared overview**. This, in turn, hindered the medical specialists in efficiently gaining a **mutual awareness** of the results from other specialties.

Although multidisciplinary consultations could be planned by means of the **orchestrating** facility, one medical specialist commented that he thought the different specialties still collaborated too little. However, he also thought that it was difficult to invite other medical specialists to multidisciplinary consultations. Based on this, the interviewee had identified a potential improvement to the system: *"Actually, you should be able to say: 'I would like to see that doctor at that specific time in my office'.* [...]. *If we allowed these systems to arrange this, this would encourage our collaboration.”* – [D-MS1]

Further, a medical specialist mentioned that the use of the **messaging** and **orchestrating** functions in the clinic led to a more digital way of collaborating. This was experienced as a negative development because face-to-face communication between healthcare professionals was seen as more valuable for discussing patient-related affairs. A medical specialist gave a crucial example in this respect: *“Certain lab results are so life-threatening that I need a phone call right away. However, currently I get these results in my messaging service with two exclamation marks behind them. This is not a problem of the EHR, but a problem of how this hospital organizes its processes.* […]. *In the past, you were called, and now you just have to find out for yourself. This can be life-threatening!”* - [D-MS3]

### Outpatient clinic E

#### Description of the outpatient clinic

Clinic E only treats patients with complex syndromes, and it consists of two specialties that focus on the same body organ. As such, these specialties have clear interfaces with each other and they consider their patients as joint patients. Before the EHR’s introduction, the clinic had already integrated all its administrative processes into the existing legacy system. The consequence of the EHR’s implementation was that all these integrated administrative processes were now divided again in the new system.

#### EHR’s facilitating influences on collaboration

Several of the interviewed medical specialists argued that the EHR could support collaboration with other specialties because the health records are **portable.** As a result, the clinic’s specialties felt better able to find specific results (such as notes) by other specialties, thereby increasing the **mutual awareness** between these specialties. Although it was argued that the **shared overview** of information could facilitate collaboration between specialties, this strongly depended on how other medical specialists used the EHR: *“Basically, it should improve collaboration – as long as the information is correct! The information is correct in the notes, but this is not always the case in the problem lists*. [...]. *I think that many patients who have had surgery have an incomplete health record. So, in terms of shared information, yes it is a plus, however, not all the information contained in the EHR is necessarily correct” -* [E-MS3].

The interviewed specialists voiced different opinions about the advantages of the **messaging** facility. Three argued that an integrated communication functionality could facilitate collaboration between different professionals. However, one could not see the added value over the earlier mail facility.

#### EHR’s constraining influences on collaboration

First, as in other clinics, several interviewees indicated that **co-located access** was hindered by not being able to simultaneously work on a health record. In this clinic, it was not so much considered as hindering collaboration between different specialties, but rather between different functional disciplines. For example, an interviewed medical specialist argued that he felt hindered when he had a joint consultation with nursing specialists. In such situations, the nursing specialist would already be working on the health record, which inhibited the physician in processing required orders through the **orchestrating** function. All the medical specialists commented that their current use of the EHR negatively influenced their ability to gain a **shared overview**. As one of them explained: “*You hear in the corridors that a number of specialties have said: 'we’re not going to do that* [entering medical histories and problem lists] *anymore' - I will not mention any names. Coincidentally, one of these departments generates half of my patients. So, if they decide to no longer enter the medical history, then I'm not going to spend half an hour per patient on it.”* – [E-MS1]. As a result, several interviewees argued that they could not trust the data contained in the **shared overview**. Consequently, the **mutual awareness** of healthcare professionals from different specialties decreased due to missing medical results.

Second, the medical specialists commented that images from two specific specialties could not be opened in the EHR as these specialties had not provided the necessary authorizations to other specialties. Accordingly, several medical specialists were hindered in efficiently developing **mutual awareness** when patients were referred from these specialties: *"It is really annoying that images of* [mentions the departments] *in particular are not yet visible in the EHR. They have blocked them from us, which is really annoying. Sometimes patients are referred, but only* [the two departments] *can see those images." –* [E-MS3]

The use made of the **messaging** and **orchestrating** affordances varied among the interviewees. Although most of them saw the advantage of **messaging** among the different specialties, one medical specialist also expressed a disadvantage in there being so many communication options during a collaboration: *“You have to make choices, you should abolish certain alternatives. Conceptually it is good that the EHR has a communication channel like that, but in practice things are really dramatic. I work with* [names a specialty], *who do everything with* ***messaging****, and I work with* [names another specialty]*, who do everything by email. Roughly 80% of my direct colleagues do everything by mail, but the other 20% use* ***messaging****. I find that really confusing.”* - [E-MS1]. One medical specialist mentioned that he did not use **messaging** because it resulted in information overload.

Further, the **orchestrating** function interfered with the collaboration between disciplinary groups. For example, medical specialists argued that some nurses refused to give daily medication to patients without an order. Related to this, there were different views on the role of verbal communication. The business manager and a medical administrator both argued that verbal communication between the different disciplines had not decreased after the introduction of **orchestrating** and **messaging.** In contrast, the medical specialists considered that verbal communication had decreased: “*Collaboration only consists of the ‘medical spam box’ (i.e.* ***messaging****).* [...]*. I find it a strange form of communication. A simple call would have worked, now the collaboration is actually very abstract.”* – [E-MS2]
